# Morphology of planktonic zoeal stages of *Palicus caronii* (Decapoda, Brachyura), identified by DNA barcoding, provides novelties to Palicoidea larval systematics

**DOI:** 10.1038/s41598-019-55412-3

**Published:** 2019-12-13

**Authors:** Giorgia Di Muzio, Rocco Mussat Sartor, Nicola Nurra, Marco Battuello, Daniela Pessani, Piero Cervella, Jose A. Cuesta

**Affiliations:** 1 Department of Life Sciences and Systems Biology, University of Torino, via Accademia Albertina 13, 10123 Torino, Italy; 2 Instituto de Ciencias Marinas de Andalucía (ICMAN-CSIC), Avda. República Saharaui, 2, 11519 Puerto Real, Cádiz Spain; 3 Pelagosphera Soc. Coop. Via U. Cosmo 17/bis 10131, Torino, Italy

**Keywords:** Zoology, Ecology

## Abstract

The zoeal development of the brachyuran crab, *Palicus caronii*, comprises two zoeal stages and the morphology is described and illustrated in detail. The zoeae were collected in plankton samples from the Southern Ligurian Sea (Western Mediterranean). Although the morphology of the larval stages of this species was unknown, a combination of characters allowed the zoeae to initially be assigned to the Palicidae, based on the previous unique known first zoeal description of one species of this family. Later, the identification of the larvae as *Palicus caronii* was confirmed through molecular analysis. The morphological features of the zoeae that characterize the Palicidae and separate them from the Crossotonotidae are confirmed. Also, the larval development comprising only two zoeal stages observed in *Palicus caronii*, the peculiar and uncommon carapace surface setation, and the presence of anterodorsal and posterodorsal sensory dorsal organs suggest that these characters could be common to the Palicoidea.

## Introduction

The larval data of the Palicoidea Bouvier, 1898 are restricted to the description of two first zoeal stages by Clark *et al*.^1^. The morphological features of the first zoea of *Crossotonotus spinipes* (De Man, 1888) and *Pseudopalicus serripes* (Alcock and Anderson, 1895) described by Clark *et al*.^1^ supported the establishment of the Palicidae Bouvier, 1898 and Crossotonotidae Moosa and Serène, 1981 as proposed by Ng *et al*.^2^ based on adult morphology, and recognized later in the systematics and classification of Brachyura by Davie *et al*.^3^.

There are few molecular data of this superfamily. However, in a molecular phylogeny of grapsoid crabs, Schubart *et al*.^4^ included in the phylogenetic analysis two palicoid species, *Palicus caronii* (Roux, 1830) and *C. spinipes*, and the results show long distances between the two species, currently considered belonging to two different families^3^.

There is only one palicid representative, *P. caronii*, in the Mediterranean Sea. This palicid is a sublittoral species inhabiting sandy bottoms with algae, and calcareous algae, as well as coralligenous hard bottoms and hard bottoms with bryozoans, in depths between 8 and 220 m but more frequently between 40 and 100 m^5,6^. Its distribution comprises the eastern Atlantic Ocean, from Annobon (Gulf of Guinea) to the Azores, including São Tomé and Principe, Ghana, Senegal, Cape Verde Islands, Canary Islands, and Madeira, and the Mediterranean Sea, from the Alboran Sea to Levantine Basin, including Balear, Adriatic, Ionian and Aegean seas^5,6^. Ovigerous females have been collected in West African waters in March, May, June, November, and December^6^, and in August and September in the Mediterranean^7^.

The generic nomenclature of *P. caronii* has been problematical. The first description of a “palicid” crab refers to *Cymopolia caronii*^8^, with an incorrect use of the generic name (already pre-occupied by the polyp *Cymopolia* Lamouroux, 1816, now Algae) that was replaced by *Palicus* Philippi, 1838^8–12^.

The systematic placement of *Cymopolia* Roux, 1830 (now *Palicus*) has been an issue especially since larval descriptions were involved. Cano^13^ described zoeae he attributed to *Cymopolia* and he assigned the genus to the Dorippidae. Later, Rathbun^14^ included *Cymopolia* within Grapsoidea. Gurney^15^ however, quoted both authors, stating that the zoea described by Cano^13^ is “unmistakably a Dorippid”. Later, Bourdillon–Casanova^16^, using the descriptions of Cano^13^, retained this genus within the Dorippidae, including it within her key to the brachyuran zoeae.

As the authors of the present study conclude that the zoeal description of Cano^13^ was based on misidentification, currently the larval development of *P. caronii* maintains the status as undescribed. Consequently, the aim of the present work is to provide the first morphological description and illustrations of plankton caught zoeal stages of *P. caronii* from the Western Mediterranean Sea, identified through DNA barcoding of the mitochondrial cytochrome oxidase subunit 1 gene (COI).

## Results

All the individuals were found all the year round in horizontal samples, showing a high peak in August 2014 with value of abundance of 0.27 ind. m^−3^ (Table 1), whereas no larvae were found in vertical samples. The larval stages identified were zoea I and II (24 and 17 specimens respectively), however, no megalops were collected. Five specimens (3 zoeae I and 2 zoeae II) were collected afterwards, exclusively for molecular analysis.

**Table 1:** Number of *P. caronii* zoeae collected during the five cruises in the three stations of the study area (see Fig. 6).

Station	Coordinates		Surveys				
Sites	Latitude	Longitude	May 2014	Aug 2014	Nov 2014	Feb 2015	Nov 2017
S1	43°29′40″	10°01′45″	2	9	1	2	1
S2	43°28′10″	10°01′55″	2	7	1	1	3
S3	43°27′10″	10°03′00″	1	15	0	0	1
Total			5	31	2	3	5
total ind m^-3^			0.04	0.27	0.01	0.02	0.02

### Molecular analysis

The COI sequence obtained from the collected zoeae did not match with any in GenBank or BOLD Systems databases. Then, the zoeal sequence was compared with that from an adult *P. caronii* specimen collected from Cartagena, Spain (that is now deposited at the CBR of ICM-CSIC, code CMHR4, and in GenBank under the accession code MN782322) by the MEGALOPADN project, showing a 99% match, with only 4 nucleotide substitutions out of 663 bp being observed.

The zoeal sequence was edited and uploaded to BOLD Systems database under the project “BMZ – Barcoding Mediterranean Zooplankton”, assigning it the Barcode Index Number ADL4122.

### Larval description

The first zoeal stage is described in detail, whereas for the second stage only morphological differences (e.g., number and/or type and position of setae) are noted.

*Palicus caronii* (Roux, 1830)

*Zoea I*

(Figures 1 and 2)

**Figure 1:**
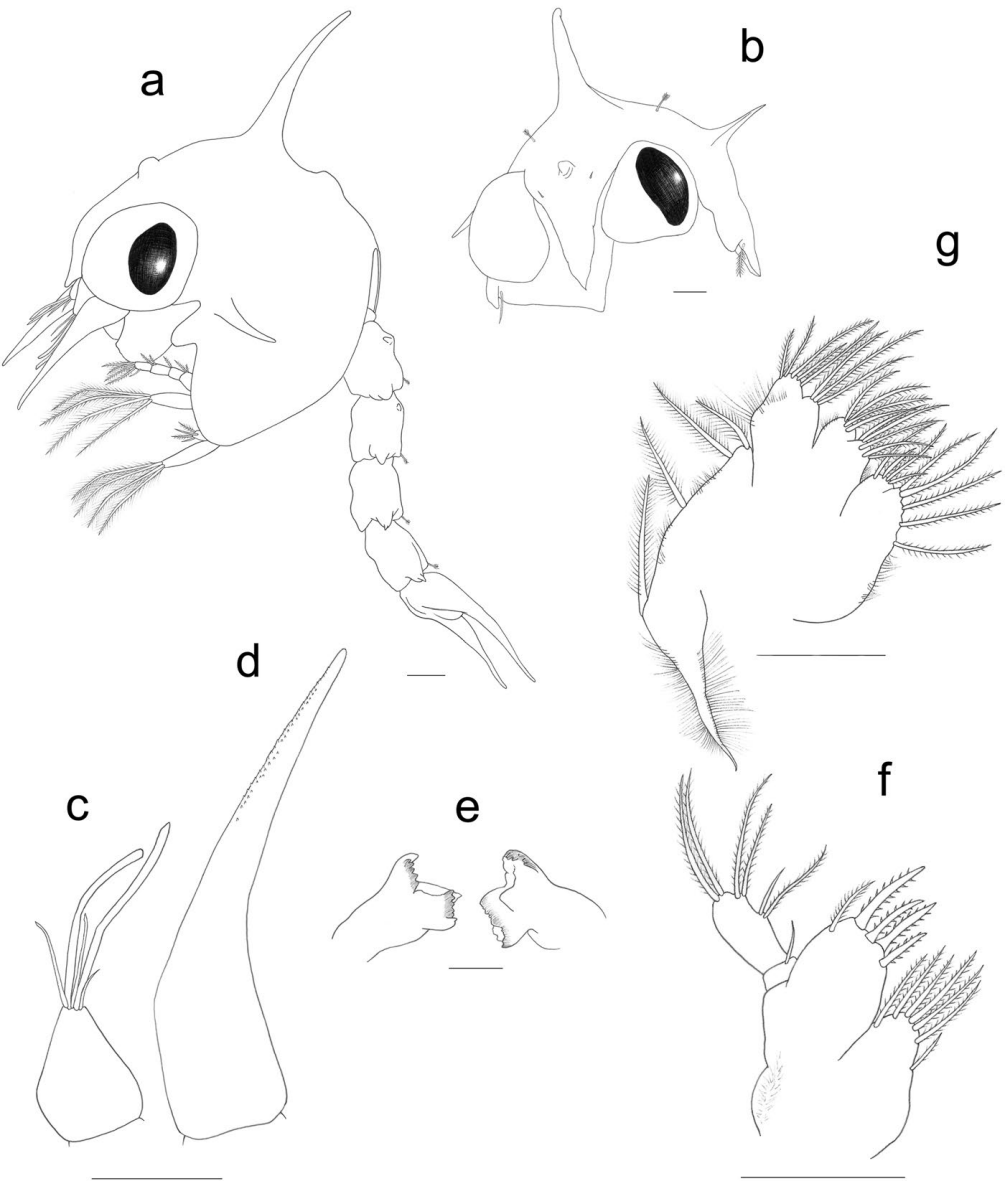
*Palicus caronii*, zoea I. (**a**) Complete specimen, lateral view. (**b**) Carapace, frontal view. (**c**) Antennule. (**d**) Antenna. (**e**) Mandibles. (**f**) Maxillule. (**g**) Maxilla. Scale bars = 0.1 mm.

**Figure 2:**
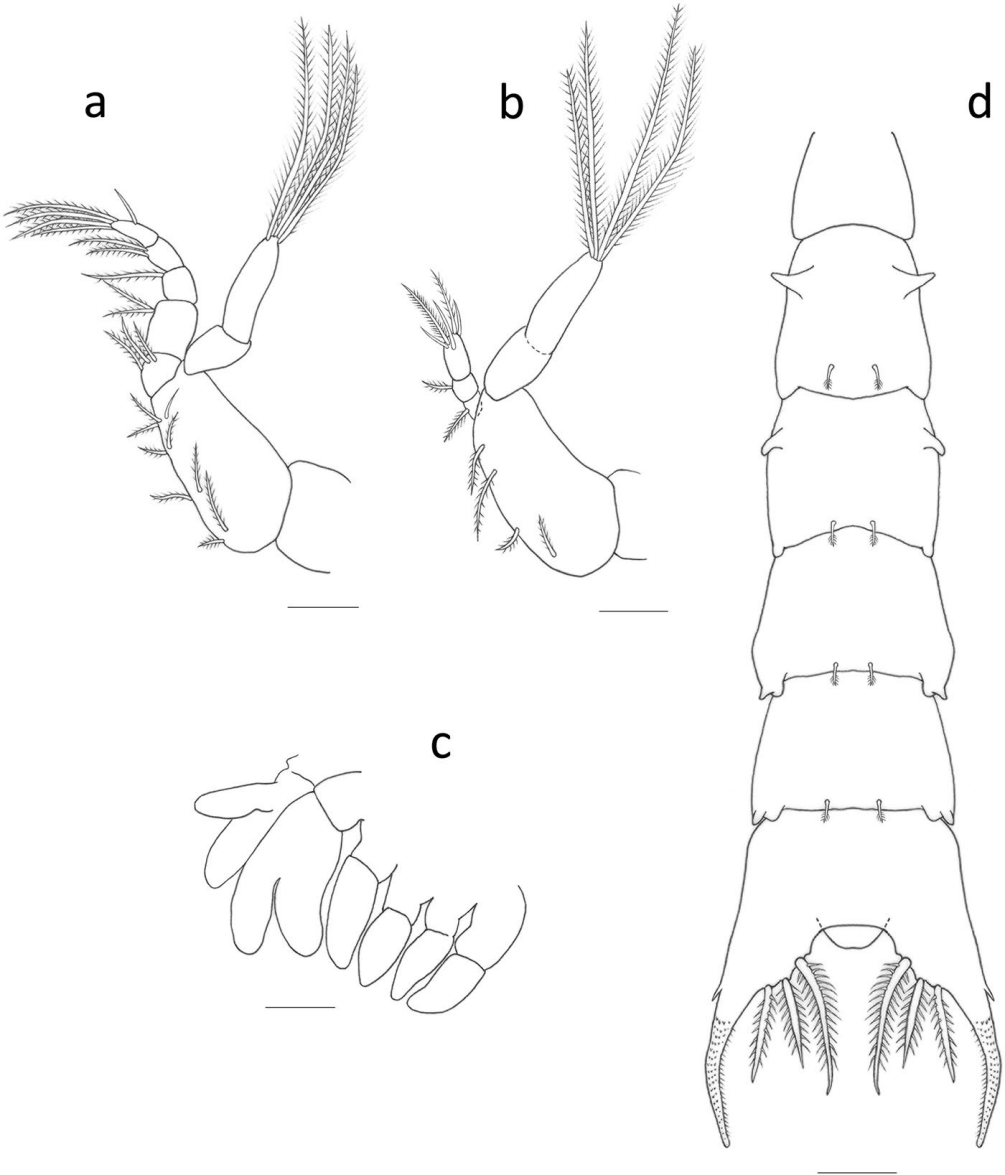
*Palicus caronii*, zoea I. (**a**) First maxilliped. (**b**) Second maxilliped. (**c**) Third maxilliped and pereiopods 1–5. (**d**) Pleon, dorsal view. Scale bars = 0.1 mm.

*Dimensions*: RDL: 1.03 ± 0.04 mm, CL: 0.66 ± 0.03 mm, RL: 0.11 ± 0.01 mm, DL: 0.45 ± 0.02 mm, CW: 0.94 ± 0.04 mm, AL: 0.34 ± 0.02 mm; n = 10.

*Cephalothorax* (Fig. 1a,b): dorsal spine moderately long, lightly curved backward and without setae; rostral spine short; lateral spines well developed; anterodorsal and posterodorsal sensory dorsal organs (SDO); one pair of posterodorsal distally plumose setae, and one pair of anterodorsal simple setae; 1 plumose anterior seta on lateroventral margin; eyes sessile. All carapace surface covered with mushroom shaped globular outgrowths.

*Antennule* (Fig. 1c): primary flagellum unsegmented with 2 long and 2 shorter and thinner terminal aesthetascs, and 1 simple seta; accessory flagellum absent.

*Antenna* (Fig. 1d): protopod well developed, long and asymmetrically distally cover with minute spinules; endopod and exopod absent.

*Mandibles* (Fig. 1e): incisor and molar process developed; palp absent.

*Maxillule* (Fig. 1f): uniramous; epipod seta absent; coxal endite with 7 setae (1 sparsely plumodenticulate + 6 plumodenticulate); basial endite with 5 setae (4 cuspidate + 1 plumodenticulate), microtrichia on proximal margin; endopod 2-segmented, with 1 simple seta on proximal segment, and 4 subterminal (1 plumodenticulate and 1 simple + 2 plumodenticulate) + 2 terminal plumose setae on distal segment; exopod seta absent.

*Maxilla* (Fig. 1g): biramous; coxal endite bilobed, with 5 + 4 plumodenticulate setae; basial endite bilobed, with 5 + 4 plumodenticulate setae; endopod unsegmented and bilobed, with 3 + 5 plumodenticulate setae; exopod (scaphognathite) margin with 4 plumose setae and a long stout plumose distal process; microtrichia present on margins of the maxilla.

*First maxilliped* (Fig. 2a): biramous; coxa without setae; basis with 9 setae (8 plumose and 1 simple) arranged 2 + 2 + 2 + 3; endopod 5-segmented with 3, 2, 1, 2 plumose, 5 (1 subterminal simple + 4 terminal plumose) setae; exopod 2-segmented with 0, 4 long terminal plumose natatory setae.

*Second maxilliped* (Fig. 2b): biramous; coxa without setae; basis with 4 plumose setae arranged 1 + 1 + 1 + 1; endopod 3-segmented, with 1, 1 plumose, 3 subterminal (2 simple and 1 serrulate) + 2 terminal (1 plumose and 1 simple) setae; exopod not clearly segmented with 0, 4 long terminal plumose natatory setae.

*Third maxilliped* (Fig. 2c): biramous bud.

*Pereiopods* (Fig. 2c): pereiopod 1 (cheliped) bilobed bud; pereiopods 2–5 uniramous buds.

*Pleon* (Figs. 1a and 2d): five pleonites present, with dorsal surface covered with a mushroom shaped, globular outgrowths; pleonite 2 with one pair dorsolateral processes directed anteriorly, pleonite 3 with one pair of dorsolateral processes directed ventrally; pleonites 3–5 with rounded posterolateral processes; pleonite 1 without setae, pleonites 2–5 with one pair of posterodorsal distally plumose setae; pleopods absent.

*Telson* (Fig. 2d): each furca distally covered with spinules, and with 1 small lateral spine; posterior margin concave with 3 pairs of serrulate setae, and a ventral medial protuberance.

*Zoea II*

(Figures 3–5)

**Figure 3:**
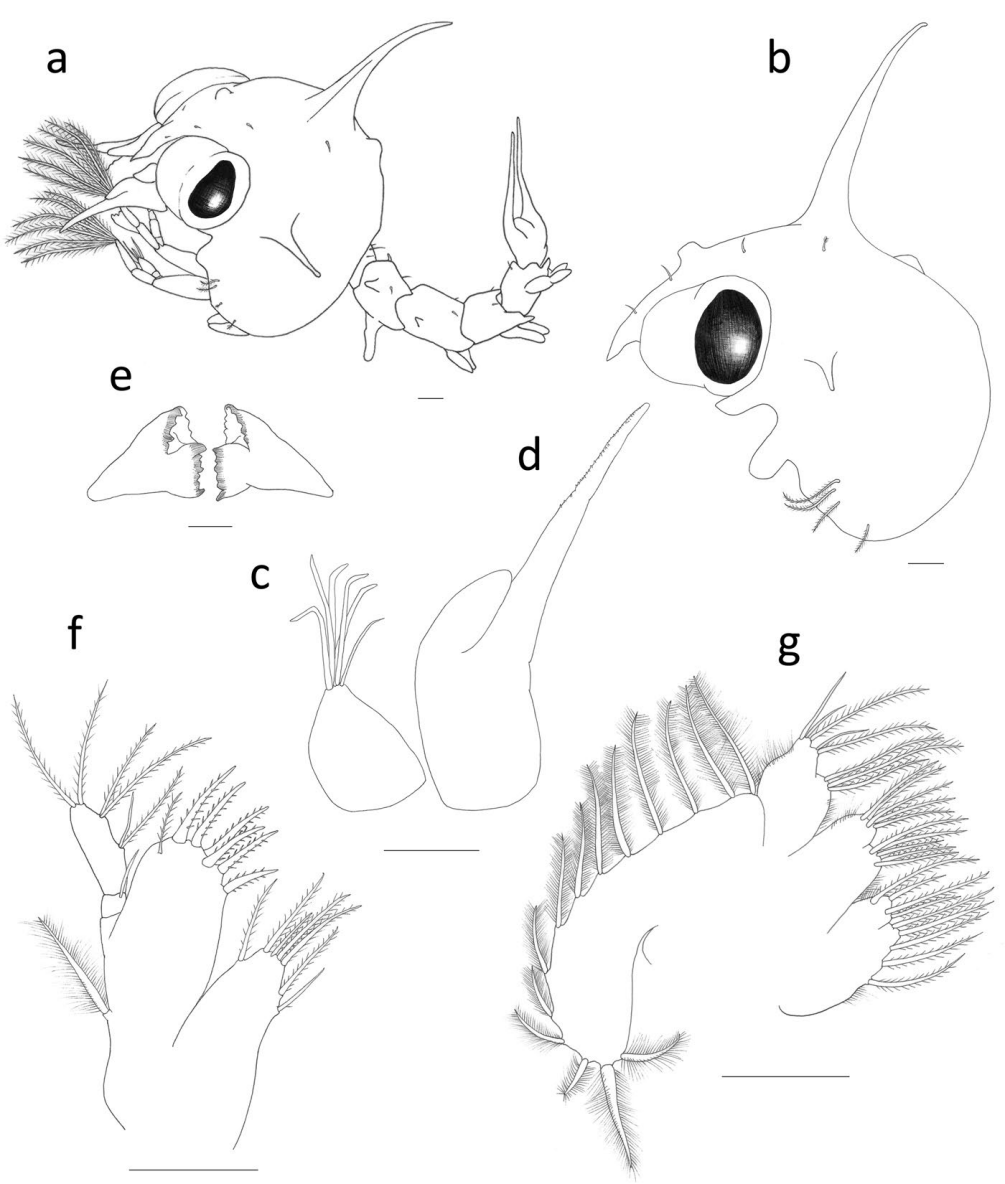
*Palicus caronii*, zoea II. (**a**) Complete specimen, lateral view. (**b**) Carapace, lateral view. (**c**) Antennule. (**d**) Antenna. (**e**) Mandibles. (**f**) Maxillule. (**g**) Maxilla. Scale bars = 0.1 mm.

**Figure 4:**
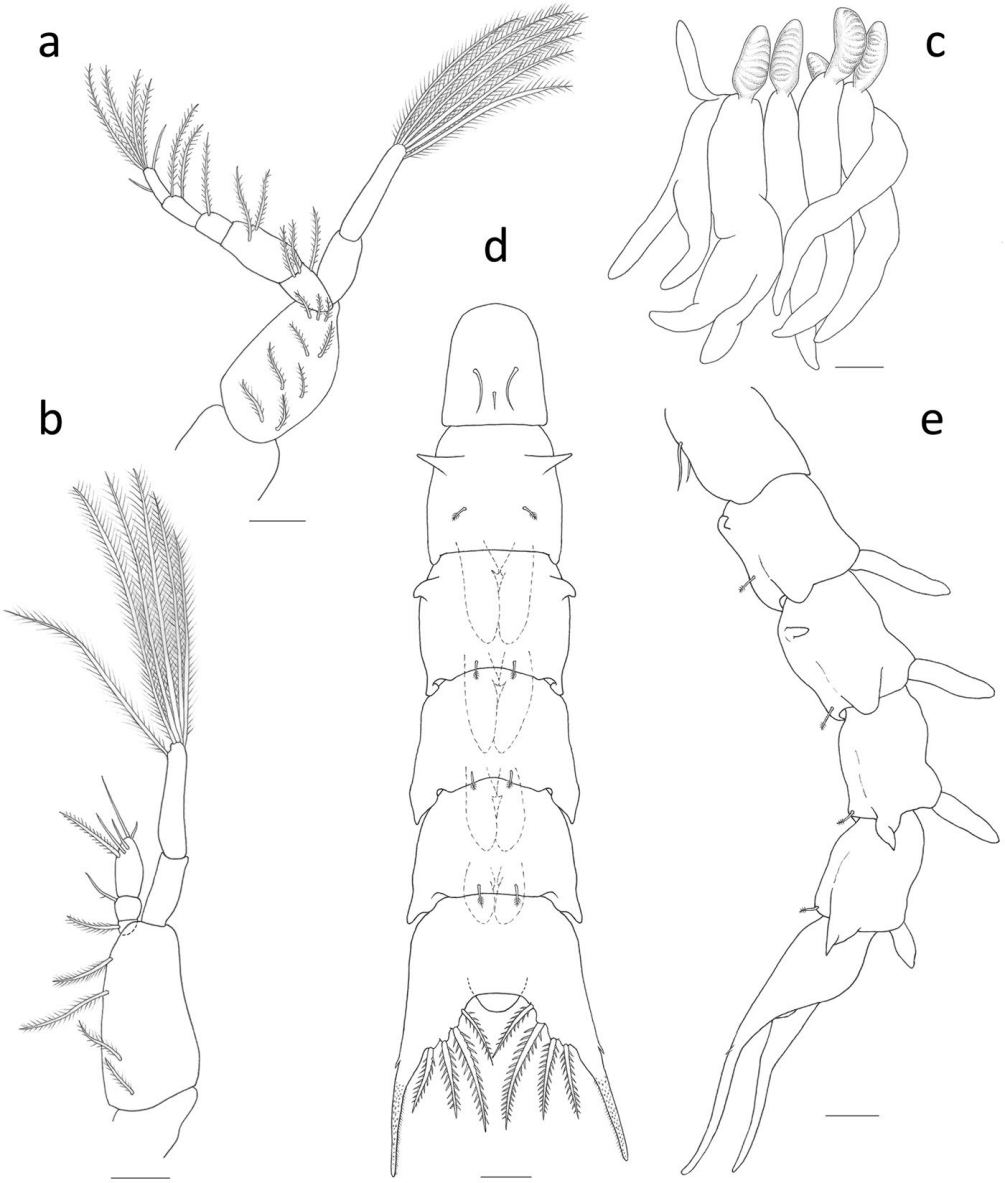
*Palicus caronii*, zoea II. (a) First maxilliped. (**b**) Second maxilliped. (**c**) Third maxilliped and pereiopods 1–5. (**d**) Pleon, dorsal view. (**e**) Pleon, lateral view. Scale bars = 0.1 mm.

**Figure 5:**
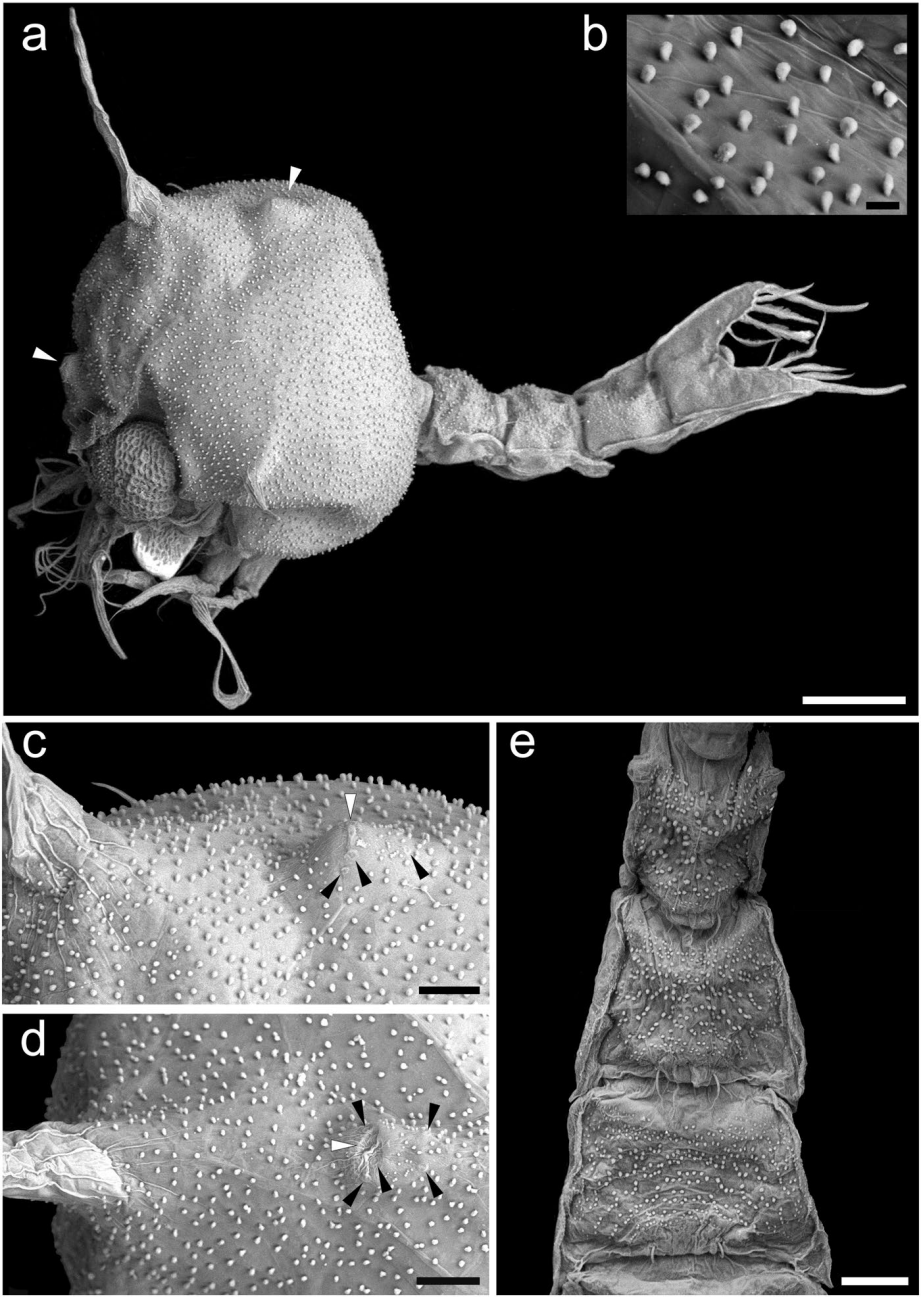
Scanning electron microscope photos of *P. caronii*, zoea II. (**a**) Complete specimen, dorso-lateral view, with anterior and posterior sensory dorsal organs (SDO) (white arrow heads). (**b**) Detail of carapace outgrowths. (**c,d**) Posterior SDO, with the central part hosting the pore (white arrow heads) and the sensory plates (black arrow heads). (**e**) Pleon (pleonites 2–4), dorsal view. Scale bars: a = 200 μm, b = 10 μm, c–e = 50 μm.

*Dimensions*: RDL: 1.42 ± 0.06 mm, CL: 0.94 ± 0.08 mm, RL: 0.19 ± 0.02 mm, DL: 0.61 ± 0.04 mm, CW: 1.27 ± 0.10 mm, AL: 0.44 ± 0.03 mm; n = 10.

*Cephalothorax* (Figs. 3a,b and 5a–d): dorsal and rostral spines slightly longer than the previous stage; two additional pairs of anterodorsal simple setae, and 3 additional plumose setae on lateroventral margin; eyes stalked.

*Antennule* (Fig. 3c): primary flagellum with 5 long terminal aesthetascs and 1 simple seta.

*Antenna* (Fig. 3d): uniramous; endopod present as elongated bud, about one-third of protopod length.

*Mandibles* (Fig. 3e): unchanged beside size.

*Maxillule* (Fig. 3f): biramous; basial endite with 7 setae (6 cuspidate + 1 plumodenticulate); endopod 2-segmented, with 1 simple seta on the proximal segment, and 2 medial (1 plumodenticulate and 1 simple) + 2 subterminal plumodenticulate + 2 terminal plumodenticulate setae on distal segment; exopod present as a long plumose seta.

*Maxilla* (Fig. 3g): basial endite with 5 + 5 plumodenticulate setae; endopod bilobed, with 3 plumodenticulate setae on inner lobe and 2 (1 plumodenticulate and 1 simple) + 3 (1plumodenticulate and 2 simple) setae on outer lobe; exopod (scaphognathite) margin with 13 plumose setae and a long stout plumose distal process.

*First maxilliped* (Fig. 4a): basis with 9 plumose setae arranged 2 + 2 + 2 + 3; endopod with 3, 2, 1, 2 plumose setae, 1 (simple) + 1 (sparsely plumose) subterminal + 4 terminal plumose setae; exopod with 0, 6 long terminal plumose natatory setae.

*Second maxilliped* (Fig. 4b): endopod 3-segmented, with 1 plumose, 1 sparsely plumose, 3 subterminal (2 simple and 1 serrulate) + 2 terminal simple setae; exopod 2-segmented with 0, 6 long terminal plumose natatory setae.

*Third maxilliped* (Fig. 4c): trilobed buds (now epipod bud present).

*Pereiopods* (Fig. 4c): elongated buds, gill buds now present.

*Pleon* (Figs. 3a, 4d,e and 5a,e): pleonite one with 3 dorsomedial simple setae; pleonites 3–5 posterolateral processes more developed; pleopods present on pleonites 2–5, biramous with small endopod buds.

*Telson* (Fig. 4d): posterior margin with 4 pairs of serrulate setae.

## Discussion

Collection of ovigerous females in good condition for identification and viable laboratory larval cultures is the traditional method for obtaining zoeae and megalopa material for morphological description. Netting plankton and identifying brachyuran larvae to species based on morphology has proved extremely difficult, if not misleading. Capturing egg bearing specimens of *P. caronii* has proved problematical. The present study, however, managed to collect plankton zoeae of this Mediterranean palicid species and confirmed its identification by DNA barcoding. This technique represents a valuable and faster method for the description of brachyuran larvae and additional characters for the appraisal of current systematics based on adult characters^17^.

Clark *et al*.^1^ were the first to describe the zoeal stages of Palicoidea species, namely *Crossotonotus spinipes* and *Pseudopalicus serripes*. Based on their descriptions of the first zoeae only, they proposed characters that allowed them to support the classification of palicoids into the Crossotonotidae and Palicidae respectively. Although the Palicoidea is a relatively small superfamily with 69 assigned species^3^, no further larval data have been published. The first zoeal stages described by Clark *et al*.^1^ were included in Table II by Clark and Cuesta^18^ of larval characters defining brachyuran families. From their table, it is apparent that the main shared familial characters included antenna type and mouthpart setation patterns and these could be considered as features at the superfamilial level. Characters that currently distinguish the Crossotonotidae and Palicidae are presence/absence of lateral spines on the cephalothorax, fourth pleonite with or without dorsolateral processes, and presence/absence of one additional small lateral spine in telson furcae. From the present study of *P. caronii*, the presence of lateral spines in the cephalothorax, the absence of both dorsolateral processes on the fourth pleonite, and the small additional lateral spine on furcae confirm that these characters are consistent within the Palicidae and distinguish them from crossotonotids.

The carapace (Fig. 5a–d) and pleon in dorsal view (Fig. 5e) of *P. caronii* zoeae have an unusual surface morphology as highlighted in the photos obtained from scanning electron microscopy, although clearly visible at optical microscopy. They are covered with mushroom shaped globular outgrowths that appear to be unique to this species. This feature is in contrast to the carapace and pleon surface morphology of *C. spinipes* and *P. serripes* which are reticulated and highly setose^1^. Such unusual surface morphology of the carapace and pleon may be considered as an additional common character of palicoid superfamily.

The zoeae of *P. caronii* present two sensory dorsal organs (SDO), one on the anterodorsal and another one on the posterodorsal regions of the carapace (Figs. 1a, 3a,b and 5a). These protuberances, although not described, are also present in the zoea I of *C. spinipes* and *P. serripes* (Fig. 1a,b and 2a,b, respectively^1^). Therefore, SDOs could be considered as another typical feature of palicoid zoeae. SEM scanning provided details of posterior SDO ultrastructure with a central pore and five or probably six surrounding sensory plates (Fig. 5c,d). This arrangement is similar to that described for other brachyuran larvae (Fig. 5b–e,k–m^19^,^20^) as a “cuticular organ complex”.

Clark *et al*.^1^ only described the zoea I morphology of two species, but the presence of biramous third maxillipeds and pereiopods buds in both cases suggested that these zoeae hatched in an advanced stage of development. The authors, however, were not able to specify the exact number of zoeal stages in palicoids larval development. The present study appears to confirm a zoeal phase with only two zoeal stages. The zoea II of *P. caronii* has all the features developed prior to the metamorphosis to megalopa (i.e. antennal endopod, third maxilliped, pereiopods, and pleopods buds well-developed). Despite of being the terminal stage, the zoea II of *P. caronii* does not possess a mandibular palp bud, in common with the same stage of *Inachus* and *Macropodia* species^18^. More descriptions of palicoid zoeal development are required to confirm whether this is also a familial character, or if it is just related to the short zoeal development with only two zoeal stages.

The description of the zoeal phase of *P. caronii* sheds some light on palicid development. Nevertheless, only the finding of the megalopa will give a complete image of larval morphology of this group. The use of DNA barcoding on plankton samples, focusing on megalopae, may be the key approach to achieve such a goal.

## Materials and Methods

### Fieldwork

**Figure 6:**
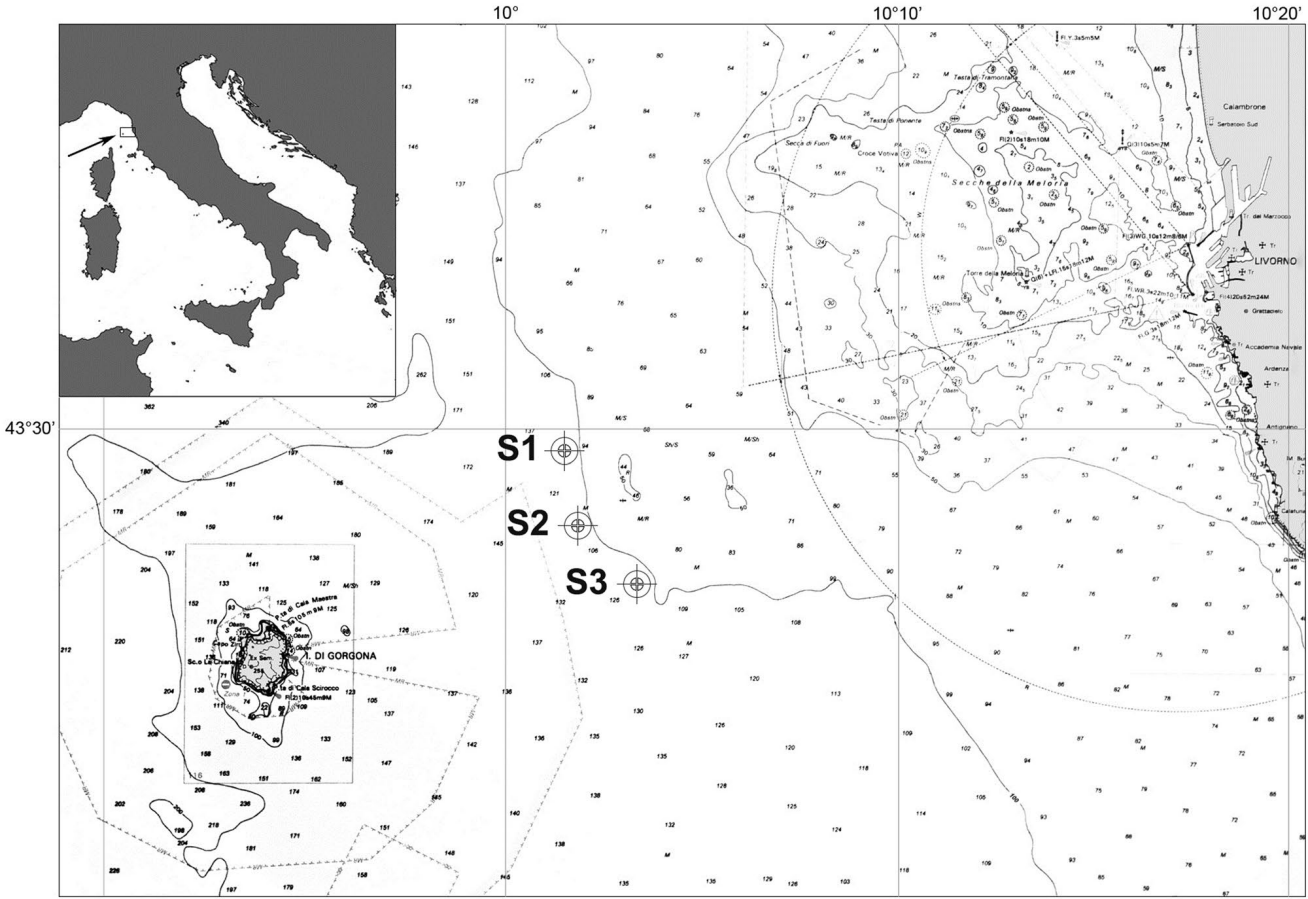
Study area: location of the three sampling sites (Stations 1, 2 and 3) in the Ligurian Sea (Western Mediterranean).

Larvae were collected in the Southern Ligurian Sea (Western Mediterranean Sea) 12.5 NM off the Tuscan coast (Italy). The sampling area is characterized by a peculiar extension of the continental shelf and shallow waters (about 100 m). The three sampling stations S1 (43°29′40″ N, 10°01′45″ E), S2 (43°28′10″ N, 10°01′55″ E) and S3 (43°27′10″ N, 10°03′00″ E) were aligned along a transect parallel to the coast as shown in Fig. 6, above bottom depths ranging from 109 to 114 m. The area was investigated for one year through seasonal sampling, making a total of four surveys: May 2014, August 2014, November 2014 and February 2015. Further sampling in the same area was performed in November 2017, in order to collect additional specimens for molecular analysis.

Zooplankton samples were collected overnight with a WP-2 standard net (ring diameter 57 cm, mesh size 200 µm), equipped with a flow meter (KC Denmark model 23.090). The sampling activity was performed through two vertical hauls (0–50 m, 50 m–bottom depth, respectively; hauling 0.7 m s^-1^) and one horizontal haul (0–2 m, approximately 15 min hauling, vessel cruising speed 2 knots).

### Sample processing

Once on board samples were immediately fixed with a solution of 4% neutralised formaldehyde (buffered with Borax) in seawater and kept in the dark^21^. During the November 2017 cruise, samples earmarked for genetic analysis were preserved in 70% ethanol. Taxa abundances are reported as individuals m^−3^.

### Molecular analysis

To recover the minimal amount of DNA required for COI amplification, total DNA was extracted from whole individuals. DNA extraction, amplification and sequencing were carried out by CCDB (Canadian Centre for DNA Barcoding), one of the main analytical nodes for the International Barcode of Life Project (iBOL), using standard procedures^22^.

The COI sequence of the zoeae was amplified using the primers ZplankF1_t1 (tgtaaaacgacggccagtTCTASWAATCATAARGATATTGG) and ZplankR1_t1 (caggaaacagctatgacTTCAGGRTGRCCRAARAATCA), a specific set of primers developed for the zooplankton, which significantly increase the average amplification success to barcode micro crustaceans^23^. The sequence will be uploaded on the Bold Systems database and a Barcode Index Number (BIN) will be assigned^24,25^.

### Morphological description

Ten specimens for each zoeal stage were measured, using a B500TPL microscope with ocular micrometer. Measurements include: rostro-dorsal length (RDL) from the tip of the rostral spine to the tip of the dorsal spine; cephalothorax length (CL) measured laterally from the frontal margin (between the eyes) to the posterior margin of the cephalothorax; rostral spine length (RL) from the base to the tip of the rostral spine; dorsal spine length (DL) from the base to the tip of the cephalothoracic dorsal spine; carapace width (CW) from tip to tip of lateral spines; antennal length (AL) from the base of the eye to the tip of the spinous process.

Following Clark and Cuesta^16^, 5 specimens for each zoeal stage were dissected under an Optika SZM2 stereo microscope and mounted in glycerine on semi-permanent slides. Drawings were made using a Leitz Dialux 22 microscope equipped with *camera lucida*.

Samples of zoea I and II have been deposited in the Invertebrate Collection of the Museo Regionale di Scienze Naturali of Torino (Italy), under the accession code MRSN Inv74.

Larval description and figures were carried out according to Clark *et al*.^26^ and Clark and Cuesta^18^. The description of the setae follows the definition and classification of Garm^27^, except for the new undescribed outgrowth found covering the surface of the carapace and the dorsal part of the pleon.

To carry out high definition images of the external and superficial morphology of the zoeae, scanning electron microscope in Low Vacuum mode was used (SEM Jeol JSM IT-300 LV), after pre-treatment of the specimens through a graded ethanol series and critical point dehydration.

### Ethical approval

This article does not contain any studies with human participants performed by any of the authors. All applicable international, national, and institutional guidelines for the care and use of animals were followed.

## Data Availability

All data generated or analysed during this study are included in this published article. Samples of zoea I and II of *Palicus caronii* have been deposited in the Invertebrate Collection of the Museo Regionale di Scienze Naturali of Torino (Italy), accession code MRSN Inv74. The COI sequence obtained from the zoeae of *Palicus caronii* was edited and uploaded to BOLD Systems database under the project “BMZ – Barcoding Mediterranean Zooplankton”, Barcode Index Number ADL4122.
